# Impact of public health interventions to curb SARS-CoV-2 spread assessed by an evidence-educated Delphi panel and tailored SEIR model

**DOI:** 10.1007/s10389-021-01566-2

**Published:** 2021-05-17

**Authors:** Bernd Brüggenjürgen, Hans-Peter Stricker, Lilian Krist, Miriam Ortiz, Thomas Reinhold, Stephanie Roll, Gabriele Rotter, Beate Weikert, Miriam Wiese-Posselt, Stefan N. Willich

**Affiliations:** 1 Institute for Health Services Research and Technical Orthopaedics, Orthopaedic Department of Medical School Hannover (MHH) at DIAKOVERE Annastift, Anna-von-Borries-Str. 1-7, 30625 Hannover, Germany; 2 Syspedics UG, Berlin, Germany; 3 Institute for Social Medicine, Epidemiology and Health Economics, Charité – Universitätsmedizin Berlin, corporate member of Freie Universität Berlin, Humboldt-Universität zu Berlin, and Berlin Institute of Health, Berlin, Germany; 4 Institute of Hygiene and Environmental Medicine, Charité – Universitätsmedizin Berlin, corporate member of Freie Universität Berlin, Humboldt-Universität zu Berlin, and Berlin Institute of Health, Berlin, Germany

**Keywords:** SARS-CoV-2, CoViD-19, Pandemic, Model, Delphi-panel, Germany

## Abstract

**Aim:**

To use a Delphi-panel-based assessment of the effectiveness of different non-pharmaceutical interventions (NPI) in order to retrospectively approximate and to prospectively predict the SARS-CoV-2 pandemic progression via a SEIR model (susceptible, exposed, infectious, removed).

**Methods:**

We applied an evidence-educated Delphi-panel approach to elicit the impact of NPIs on the SARS-CoV-2 transmission rate R_0_ in Germany. Effectiveness was defined as the product of efficacy and compliance. A discrete, deterministic SEIR model with time step of 1 day, a latency period of 1.8 days, duration of infectiousness of 5 days, and a share of the total population of 15% assumed to be protected by immunity was developed in order to estimate the impact of selected NPI measures on the course of the pandemic. The model was populated with the Delphi-panel results and varied in sensitivity analyses.

**Results:**

Efficacy and compliance estimates for the three most effective NPIs were as follows: test and isolate 49% (efficacy)/78% (compliance), keeping distance 42%/74%, personal protection masks (cloth masks or other face masks) 33%/79%. Applying all NPI effectiveness estimates to the SEIR model resulted in a valid replication of reported occurrence of the German SARS-CoV-2 pandemic. A combination of four NPIs at consented compliance rates might curb the CoViD-19 pandemic.

**Conclusion:**

Employing an evidence-educated Delphi-panel approach can support SARS-CoV-2 modelling. Future curbing scenarios require a combination of NPIs. A Delphi-panel-based NPI assessment and modelling might support public health policy decision making by informing sequence and number of needed public health measures.

**Supplementary Information:**

The online version contains supplementary material available at 10.1007/s10389-021-01566-2.

## Background

COVID-19 (SARS-CoV-2) pandemic is a major global health threat and has caused more than 34 million detected cases of COVID-19 disease and claimed > 1 million lives worldwide as of end of September 2020 (WHO 2020).

In the absence of COVID-19 specific therapies and a SARS-CoV-2 vaccination, as well as when vaccination is incomplete, only public health interventions can reduce the impact of COVID-19 on mortality, morbidity, and the associated resource use. A key focus in managing the COVID-19 pandemic is to mitigate the epidemic peak, also known as ‘flattening the (epidemic) curve’.

Mitigation or ideally extinction of a pandemic is currently restricted to non-pharmaceutical interventions (NPI) only. Several public health intervention measures to control COVID-19 are available, including testing and isolation, social distancing, school closures, appeal for improved hand hygiene, shielding of risk populations, self-isolation, working from home, and different forms and levels of lockdown (office, restaurants, shopping).

Despite the as yet small evidence about age profile of susceptibility and infectivity, scarce information on frequency of super-spreading events, little data on transmission-rate in households, and the not yet scientifically described contribution of asymptomatic individuals to transmission (Hu et al. 2020; Sun and Viboud 2020), several parameters for modelling are available from different countries and settings (ECDC 2020; Meehan et al. 2020).

A wide range of models have been developed with different elements and levels of modelling details (Dehning et al. 2020; Ferguson et al. 2020; Koo et al. 2020; Lourenco et al. 2020; Wu et al. 2020). Modelling objectives cover the following areas: providing initial estimates of the SARS-CoV-2 reproduction rate with and without intervention implementation, assessing regional or global spread, and quantifying the severity and burden of COVID-19 (Meehan et al. 2020). Most models are based on the classic structure of SEIR covering susceptible (number of susceptible individuals), exposed (number of exposed individuals), infectious (number of infectious individuals) to removed (number of removed individuals either by recovery with full immunity, by immunisation, by individuals being deceased, or by isolation) (Brauer and Castillo-Chavez 2012). Often *R* is defined as recovery only; however, Kermack and McKendrick (Kermack and McKendrick 1927) already introduced the definition “removed by recovery or death”..

SARS-CoV-2 models have applied certain sets of measures such as case isolation in the home, (voluntary) home quarantine, social distancing of those over 70 years of age, social distancing of the entire population, closure of schools and universities (Ferguson et al. 2020; Huang et al. 2014). Multiple measures, also described as a targeted layered containment (Halloran et al. 2008), might be individually ineffective but effective in combination with other measures (Eubank et al. 2020). Hence, public health measures might have an impact on each other and also the impact of order of implementation might be of relevance.

The objectives of this study were to use a Delphi-panel based assessment on the effectiveness of different COVID-19 specific prevention measures in order to retrospectively approximate, and to prospectively predict SARS-CoV-2 pandemic progression via a SEIR model selecting different NPI scenarios in Germany. Access to the model is intended to be public domain.

## Methods

### Delphi approach

We applied an evidence-educated Delphi-panel approach to elicit the impact of NPIs being discussed in Germany. The Delphi panel was comprised of ten public health experts experienced in systematic reviews from Charité – Universitätsmedizin Berlin and Medizinische Hochschule Hannover (epidemiologists, health system researchers, virologists, experts for hygiene, biometricians, and social medicine physicians). At the end of May 2020, experts contributed about 1 day of literature review-approximated research to each measure, and, based on this knowledge, had to provide both a first estimate of potential efficacy in reducing R_0_ (in %) of the one assigned NPI measure and a first educated suggestion on compliance of assigned measure in Germany (in %) as well as a summary of the key search results. Covered NPI measures were:

- using personal protection masks (cloth masks or other face masks),
- appeal for improved hand hygiene,
- test and isolate,
- ban of large public events (> 1000 participants),
- closure of schools and universities,
- working from home,
- social distancing of the entire population (with the components “contact reduction” and “keeping distance”),
- closure of restaurants,
- closure of non-essential stores.

Subsequently, experts’ opinions on efficacy and compliance under real-life conditions were gathered in a two-stage Delphi process designed to combine each opinion into an average consensus value (McKenna 1994). Initial expert estimates were discussed in an online face-to-face group discussion, and efficacy and compliance judgements were collected from all experts. First-stage mean group values were circulated after 1 week for a subsequent educated feedback in order to provide opportunity for changing opinions. Mean values of the new votes of the second stage were taken as the final efficacy and compliance values. Effectiveness was defined as the product of efficacy and compliance.

### Model

We employed a discrete, deterministic SEIR model on a daily basis for a period of less than 1 year without considering crude or Covid-19 specific death rates (Kermack and McKendrick 1927). The effectiveness of non-pharmaceutical intervention measures was modelled as a temporary reduction of the (basic) transmission rate β. We distinguished between *scalable measures* that do not depend on the number of infected individuals and *resource-dependent measures* that depend on a given capacity (e.g., number of test-and-isolate per day) divided by the number of infected individuals I(t) which are those accessible by the measure.

The combination of all individual NPI measures could theoretically reach 100% resulting in the extinction of the pandemic course. However, even in a setting where a wide range of NPIs had been implemented comprehensively and restrictively such as in China, an extinction could not be observed (Bi et al. 2020; Lai et al. 2020). Hence, we addressed a potential bias in overestimating combined effectiveness of NPIs with an adjustment differentiating between scalable measures and resource-dependent measures.

For each NPI, we imputed an officially-announced coming-into-force date (either obtained on federal level or if not available approximated via Länder information), an estimated advanced uptake period of the NPI, and a decline of compliance after reaching maximum compliance, as well as a spill-over effect of each NPI addressed as a residual-compliance > 0 after the announced coming-out-of-force date.

The following base case assumptions were chosen for obtaining best fit with reported data:

-  R= 3.8 (Liu et al. 2020b)^1^
- latency period λ = 1.8 days (Guan et al. 2020; Jing et al. 2020; Liu et al. 2020a)^2^
- duration of infectiousness δ = 5 days (Jing et al. 2020; Singanayagam et al. 2020)^3^
- infection rate β = *R*/ δ = 0.76/day
- share of immunity α = 15% of the total population
- estimated ratio of unreported cases = 5
- reporting delay = 8 days.

All scalable NPIs except “test-and-isolate” are employing a function of gradually being introduced, waning out over time except for “ban of large events”. All time points for NPI measure initiation, NPI measure duration, and NPI measure relief can be changed individually. The NPI “test-and-isolate” was considered to be resource-dependent according to the resource being available at public health departments in the first quarter of 2020. We estimated the minimum initial capacity for the “test and isolate” strategy in February based on available medical personnel of local and regional public health departments, resulting in a capacity of 1000 incident cases per day in Germany to be approached, checked for further contacts, and followed up.

### Validation

The model was validated via a simple SEIR model in order to check reproducibility of standard disease transmission and peak behavior. Validation obtained identical results to an Excel-based model. Published German data were used to approximate SEIR model input parameters and the empiric course of the COVID-19 epidemic was based on Johns–Hopkins data (https://coronavirus.jhu.edu/data). When validating the contribution of the latency period, the variation of the parameters resulted in expected real-life scenarios providing adequate face validity.

### Model data population

All Delphi effectiveness data were introduced into the model base case following the time order obtained either directly from published German announcements or indirectly via statements of decision makers or media coverage.

Figures are presented as total estimated cases (including not reported cases).

### Sensitivity analysis

Two-dimensional sensitivity analyses were performed for the basic reproduction number R, latency period λ, the general compliance level W and the offset O of the coming into force of the mitigation measures.

### Patient and public involvement

It was not appropriate or possible to involve patients or the public in the design, or conduct, or reporting, or dissemination plans of our research.

## Results

**Fig. 1:**
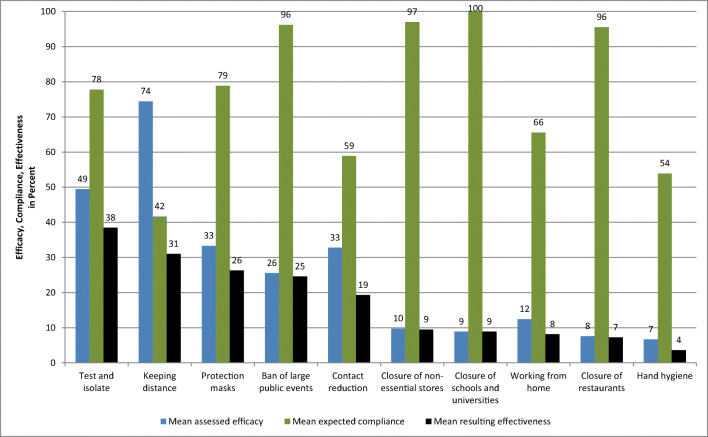
Results of the Delphi-panel assessments. *Blue*: mean estimated efficacy being achievable. *Green* mean expected compliance. *Black*: resulting mean effectiveness (i.e., product of efficacy and compliance)

Delphi-based estimation attributed the highest efficacy to the NPI “keeping distance” with a reduction of R of 74%, followed by “test and isolate” of 49%, and “wearing protection masks (cloth masks or other face masks)” of 33%. Lowest efficacy was estimated for “hand hygiene” at 7%, preceded by “closure of restaurants” at 8% (Fig. 1a). Compliance for “keeping distance “was estimated at 42%,” test-and-isolate” 78%, and “wearing protection masks” 79%; “hand hygiene” was estimated to achieve compliance of 54%, and “closure of restaurants” of 96% (Fig. 1).

**Fig. 2:**
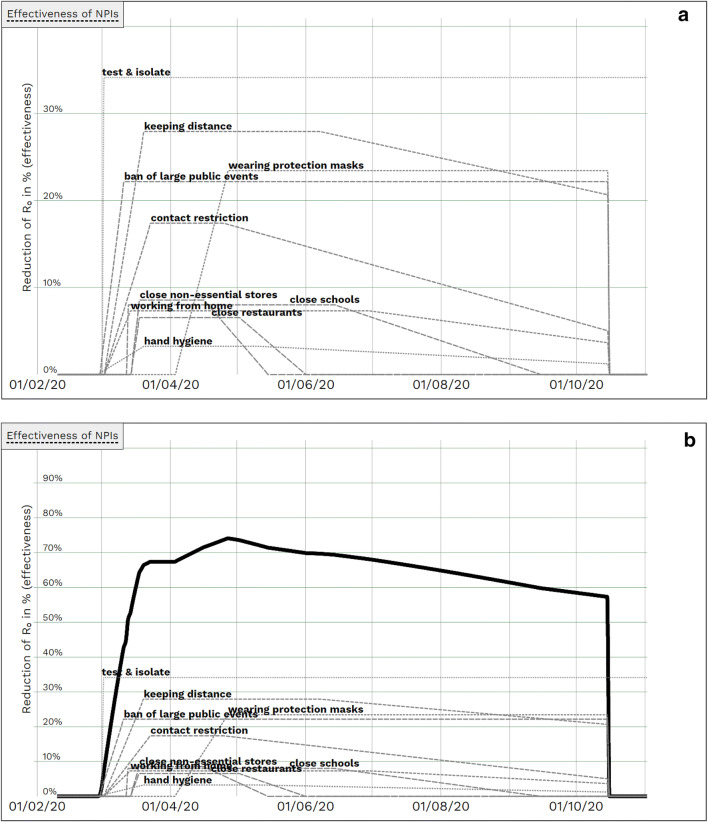
All NPIs with time-dependent effectiveness curve (due to time-dependent compliance rates and coming into force dates**)**. **a** All NPIs with time-dependent effectiveness curves (*dotted/dashed lines* represent course of effectiveness of NPIs over time incorporating time-dependent coming into force dates and estimated fading-in and waning phases). **b** All NPIs with time-dependent effectiveness curves and cumulated effectiveness curve (*solid black*). Please note the different *y*-scale compared to **a**

Collection of information obtained either directly from published German announcements or indirectly via statements of decision makers or media coverage resulted in NPI-specific effectiveness curves based on coming into force dates of NPIs and estimates on preliminary fading in and waning, with “test and isolate” being continuously available. (Fig. 2a).

**Fig. 3:**
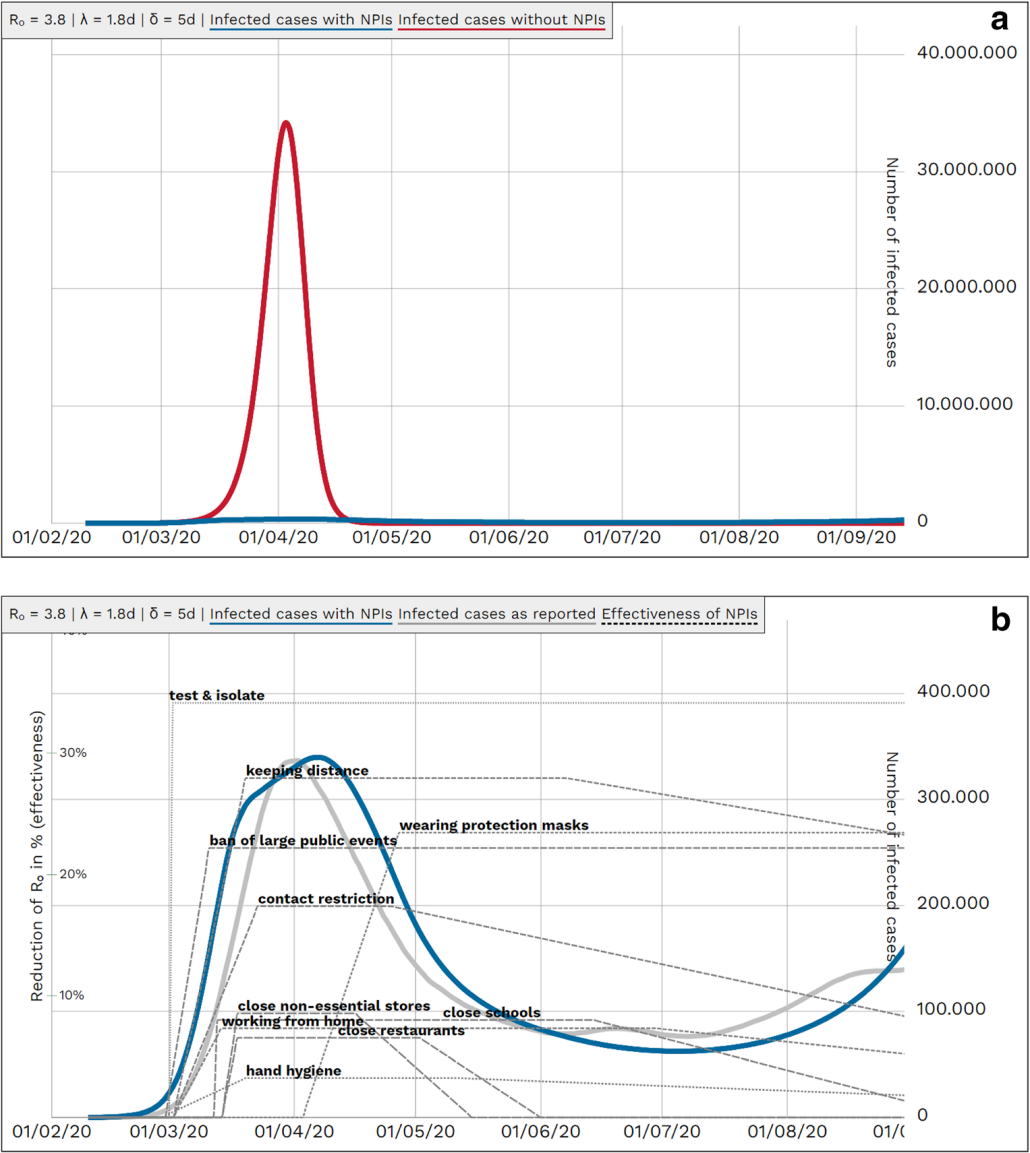
Reported and modelled numbers of infected cases from February to September 2020 in Germany. *R*_0_: basic reproduction number, *λ*: latency period, *δ*: duration of infectiousness, *NPI*: non-pharmaceutical interventions**.** **a** Modelled course of COVID-19 pandemic in Germany until September 2020 with NPIs (*blue*) and without NPIs (*red*). *Blue and red lines* represent simulated case numbers. **b** Reported (*grey*) and simulated (*blue*): course of COVID-19 pandemic with single NPIs in Germany until September 2020. *Grey line* is based on reported data (7-day moving average), assuming five times as many unreported cases as reported cases and reporting delay of eight days. Left *y*-scale — effectiveness of measures; right *y*-scale — population

When populating the model with the Delphi-based effectiveness results and applying reported German coming-into-force periods, the German course of reported COVID-19 incident infected cases could be approximated when applying a global scaling factor Φ of 0.9 for all compliances. (Figs. 3a and b).

**Fig. 4:**
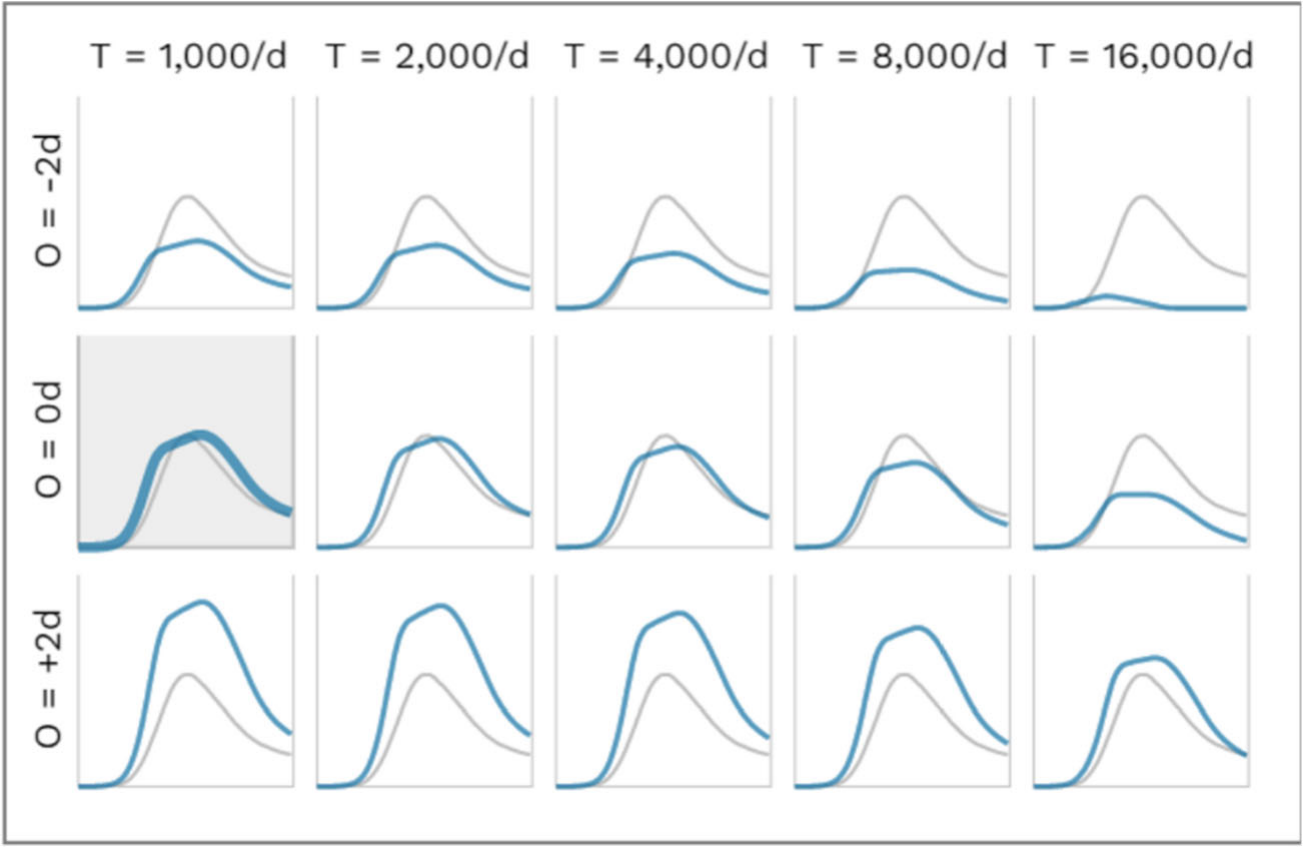
Retrospective counterfactual scenarios with variation both of test and isolation capacity and start time in order to contain spread of SARS-CoV-2

We simulated counterfactual scenarios in which “test-and-isolate” is the only mitigation measure. No peak in infected cases was seen only at a threshold capacity of being able to test, manage, and isolate more than 16.000 new identified cases per day, resulting in a containment of the spread of SARS-CoV-2 in Germany. (Fig. 4).

**Fig. 5:**
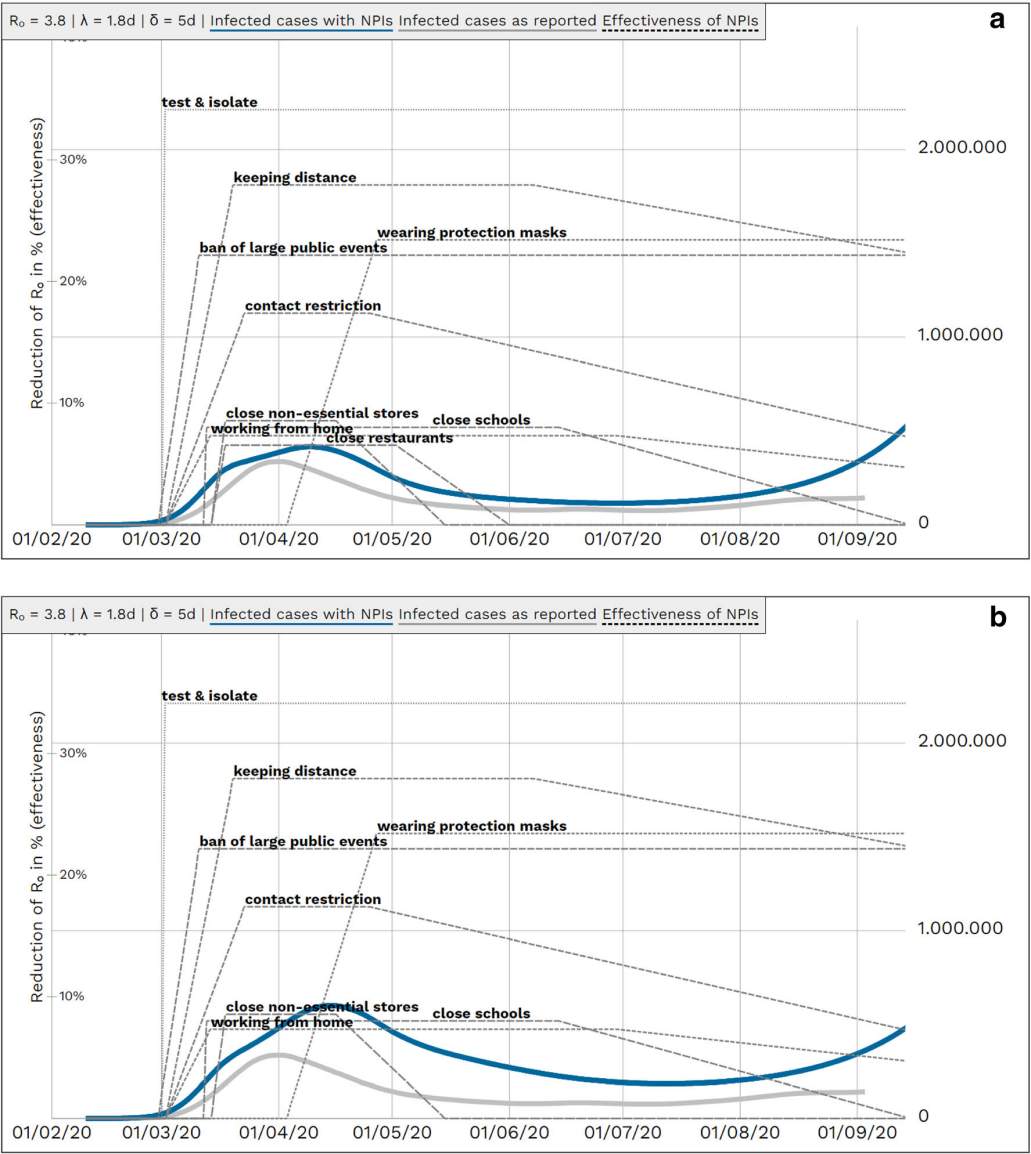

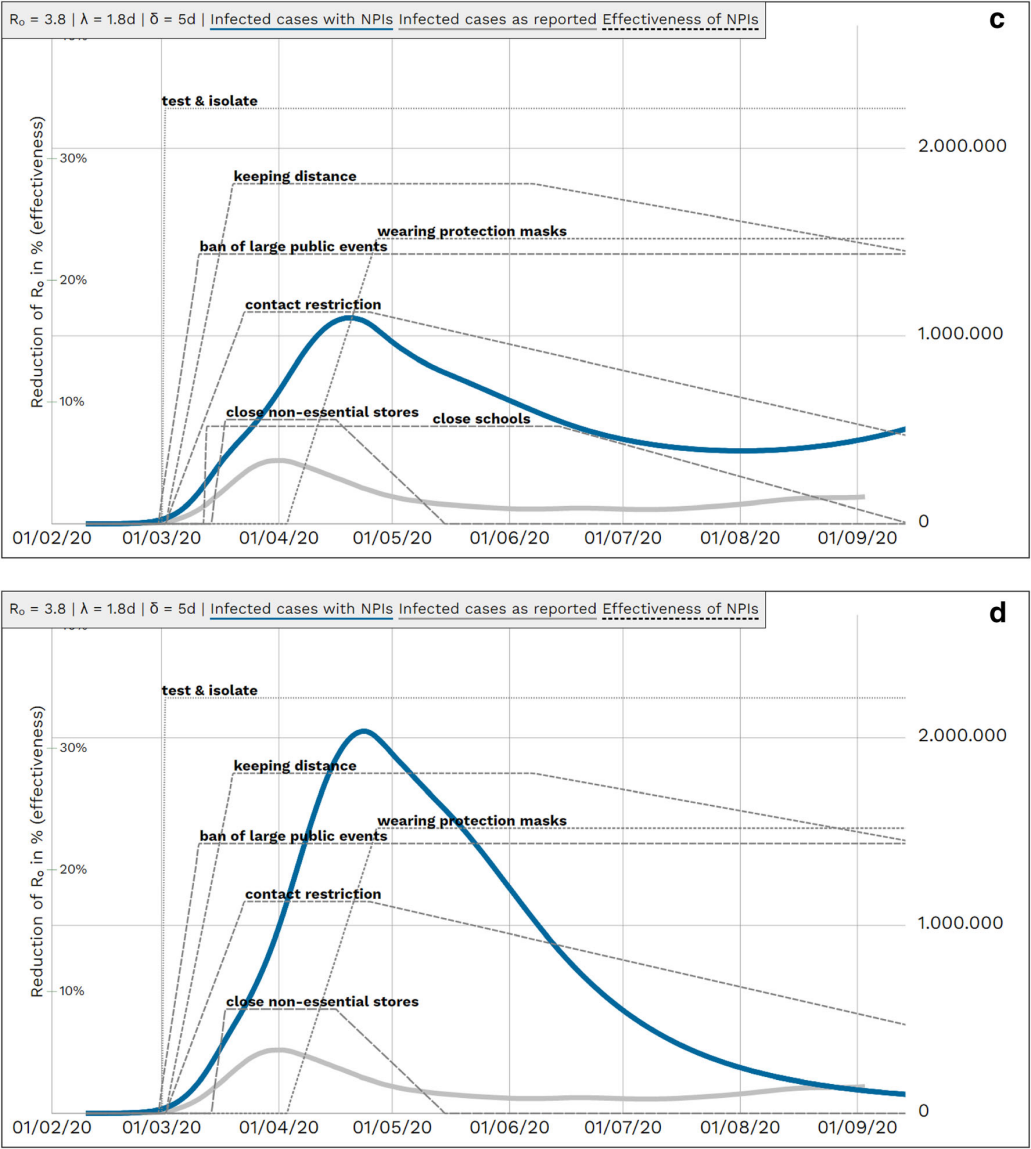
Retrospective counterfactual scenarios modelling the effect of lower impact measures. *R*_0_: basic reproduction number, *λ*: latency period, *δ*: duration of infectiousness, *NPI*: non-pharmaceutical interventions**.** *Blue line*: simulated case numbers, *grey line*: case numbers as reported, *dotted lines*: effectiveness of NPIs. Left *y*-scale — effectiveness of measures; right *y*-scale — population. **a** All NPIs (as in Fig. 2b) excluding the NPI “hand hygiene”. **b** As in **a** additionally excluding the NPI “close restaurants”. **c** As in **b** additionally excluding the NPI “close schools”. **d** as in **c** additionally excluding the NPI “working from home”

In a further hindsight approach, we simulated counterfactual retrospective scenarios successively turning off the lower impact NPIs “hand hygiene”, “close restaurants”, “close schools”, and “working from home”. (Figs. 5a-d).

**Fig. 6:**
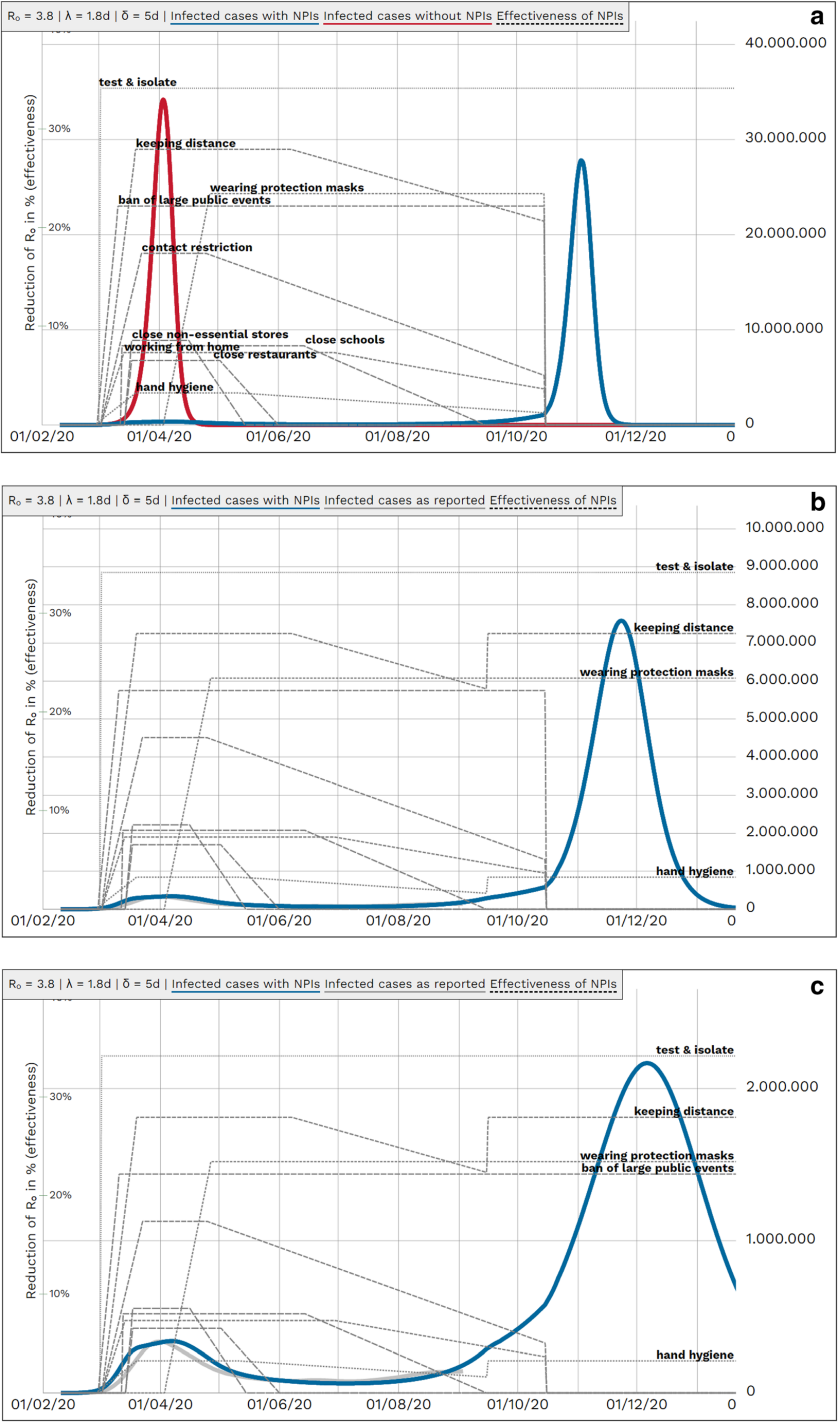

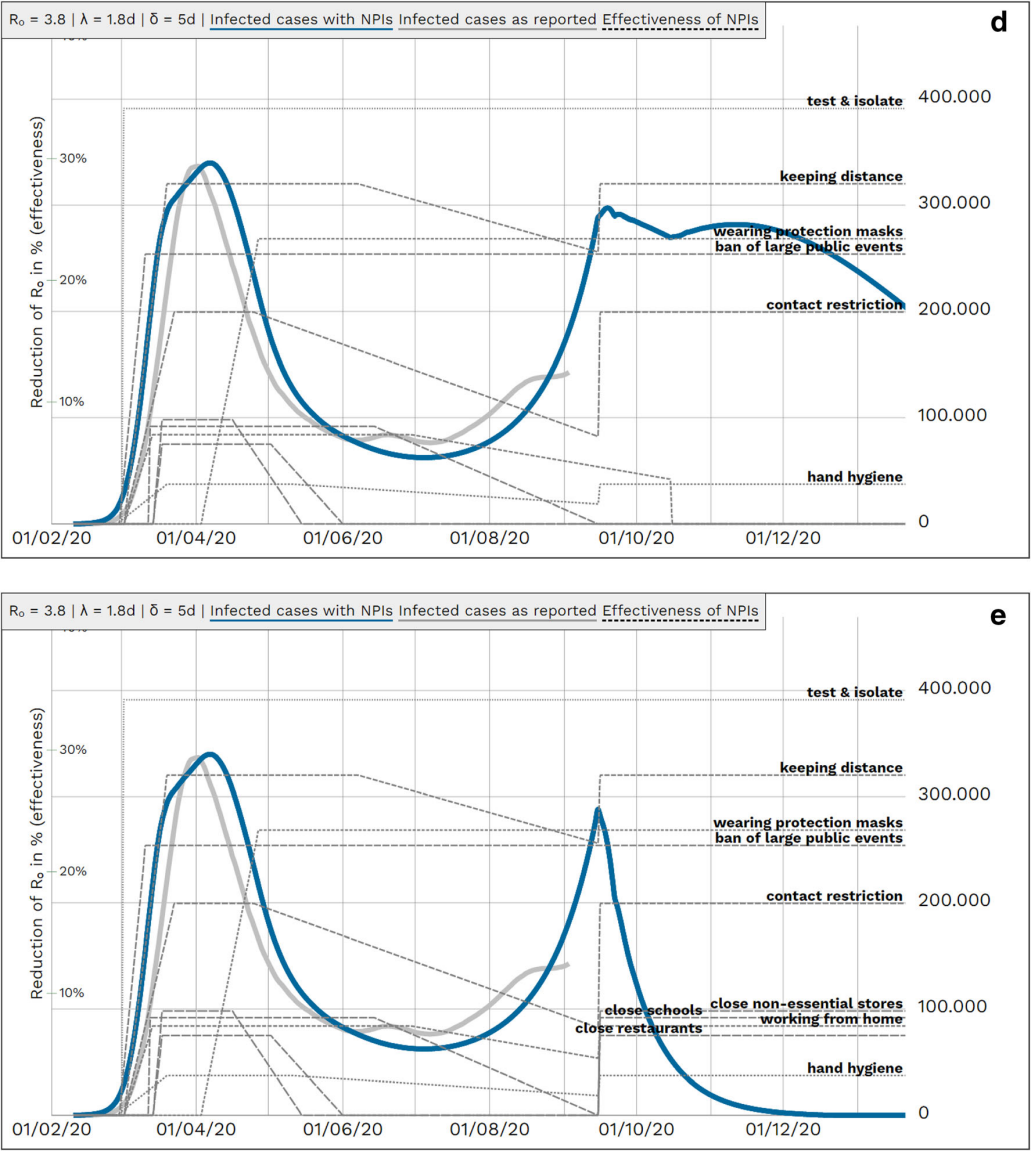
Prospective scenarios estimating course of pandemic with renewed coming into force of NPIs at September 15, always maintaining “test and isolate”. *R*_0_: basic reproduction number, *λ*: latency period, *δ*: duration of infectiousness, *NPI*: non-pharmaceutical interventions. *Blue line*: simulated case numbers, *grey line*: case numbers as reported, *dotted lines*: effectiveness of NPIs, Left *y*-scale — effectiveness of measures; right *y*-scale — population. **a** Simulated course of COVID-19 pandemic in Germany until end of 2020 if all measures were turned off at October 15 (*blue line*). **b** Renewed coming into force of German AHA-rule (“keeping distance”, “hand hygiene”, “wearing protection masks”). **c** Renewed coming into force of the German AHA-rule as in **b** and additionally “ban of large public events”. **d** Renewed coming into force of NPIS as in **c** and additionally “contact restriction”. **e** Renewed coming into force of all NPIs. [For **b** to **e**, please note different *y*-scale!]

When assuming no further measures after mid-August 2020, a second peak would have arrived in October 2020 (Fig. 6a). When combinations of NPIs were reinforced after a supposed end of German NPIs mid of August (immediately and at full, i.e., not decaying effectiveness) the following results could be observed (Figs. 6b-e): When applying the German AHA^4^ rule (i.e., “keeping distance”, “hand hygiene” and “wearing protection masks”) and “ban of large public events”, i.e., measures considered less economically detrimental, about 2 and 3 million cases would be expected (Fig. 6c). Reinforcing the German AHA rule as well as “ban of large public events” and “contact restriction” would have controlled the outbreak at a level of 300.000 cases (Fig. 6d). When all NPIs were reinforced immediately, the second outbreak would have been fully controlled.

### Sensitivity analysis

**Fig. 7:**
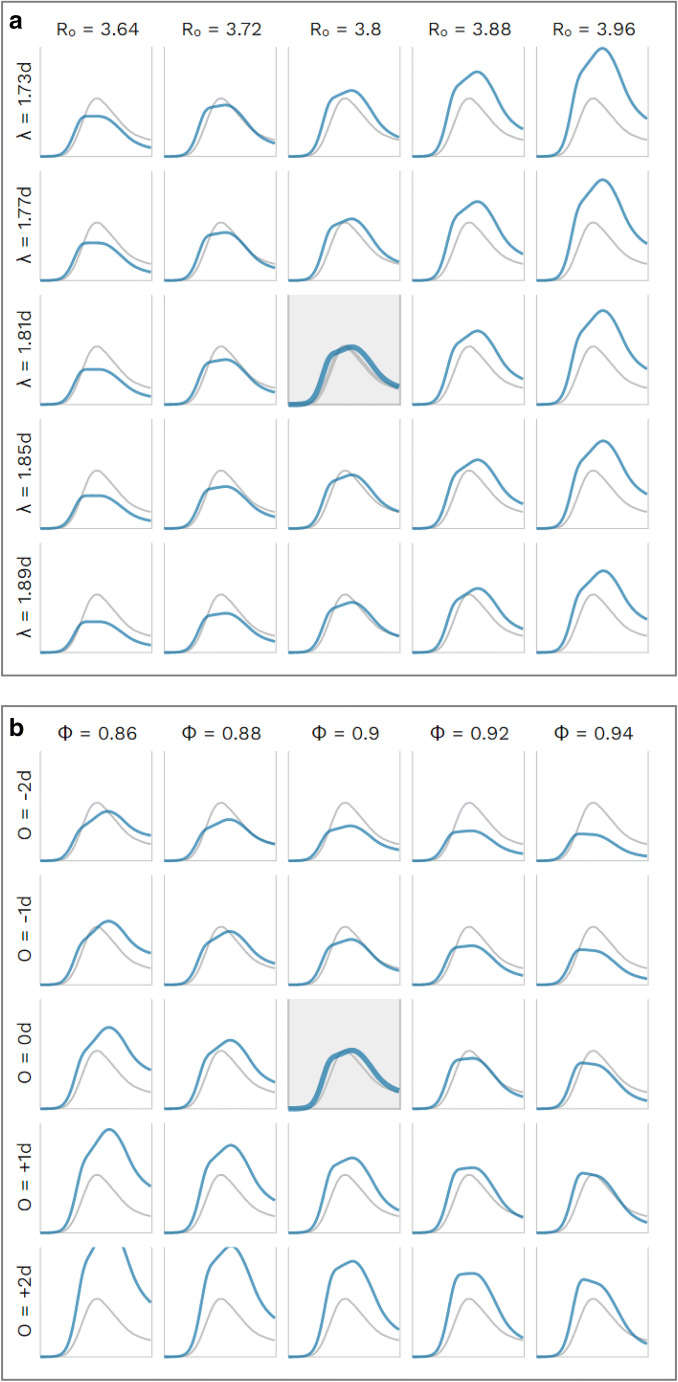
Sensitivity analysis for **a** basic reproduction number R_0_ and latency period λ and **b** global scaling factor Φ and days offset O of the coming into force of the mitigation measures. *Blue line*: simulated case numbers, *grey line*: case numbers as reported. **a** Sensitivity analysis for R = 3.8 ± about 2% and λ = 1.8 ± about 2% days; all other parameters kept fixed (including mitigation measures). The highlighted chart corresponds to our base case with R = 3.8 and λ = 1.8 days. *R*_0_: basic reproduction number, *λ*: latency period**. b** Sensitivity analysis for global scaling factor Φ = 0.9 ± 2% and days offset O of coming into force of NPIs (O = 0 d: without offset, as in Fig. 1b, O = +1 d: 1 day later, O = −1 d: 1 day earlier), all other parameters kept fixed. The highlighted chart corresponds to our base case with Φ = 0.9 and O = 0 days

In the two-dimensional sensitivity analyses the a) basic reproduction number R and latency period λ and b) the general compliance level W and the offset O of the coming-into-force of the mitigation measures are varied. (Fig. 7a-b).

## Discussion

We examined whether a Delphi-panel-based assessment of the effectiveness of different COVID-19 specific public health prevention measures could serve as a basis for populating a SEIR model. We also retrospectively approximated the course of a range of mitigating scenarios with a potentially reduced economic impact in Germany. Finally, we examined a range of likely SARS CoV-2 transmission scenarios depending on enforced non-pharmaceutical interventions (NPI) that could mitigate the intensity of the pandemic following the initial outbreak in Germany. Our modelling approach was intended to serve as a public health decision tool in order to support decision-makers in the regional and national healthcare settings, and will be made accessible on a public domain basis.

A Delphi-panel-based assessment was chosen as it allowed for combining evidence-based knowledge and educated assumptions, with the purpose to collect opinions on effectiveness of a wide range of NPIs in a timely manner (Avella 2016). As several SARS-CoV-2 transmission details are subject for research, we based our model on available evidence with regard to COVID-19 characteristics (Anderson et al. 2020; Hu et al. 2020; Sun and Viboud 2020).

The Delphi-panel process agreed highest efficacy estimates for “test-and-isolate” followed by “keeping distance”, “wearing personal protection masks”, and “ban of large public events”. Lowest impact was agreed for “closure of non-essential stores”, “closure of restaurants”, and “hand hygiene”. This is in line with the findings of a recent longitudinal retrospective analysis of the RKI based on data from 37 countries identifying restrictions on gatherings, mask-wearing requirements and school-closing requirements as leading contributing NPIs (Pozo-Martin et al. 2020). In our model, lifting measures without immunisation being available induced a more or less pronounced second wave with a peak size depending on the maintained measures and their overall enforcement or compliance. A combination of at least five measures with a perceived lower economic impact is needed to achieve a sustainable control of SARS-CoV-2 spread.

In the beginning and end of a pandemic situation, case detection and contact reduction (i.e., “test-and-isolate”) is a strong contributor to managing the pandemic’s course (Lai et al. 2020). When having been able to intervene early enough, such as the first official patient being identified in Munich (the so-called Webasto cluster), test-and-isolate was fully effective (Wolfel et al. 2020). Intensive testing and case-based interventions have so far formed the centrepiece of control efforts in some places, including Singapore and Hong Kong (Anderson et al. 2020). However, the contribution of this measure is linked to capacity issues. When cases were being introduced on a delayed basis as with infected ski-vacationers returning to Germany and large public events such as Karneval (carnival in Cologne) being visited by regional population clusters in March, even within a range of a simulated 1000 to 8000 test capacity per day and without any other measures being in place, the pandemic could not have been contained. However, when introduced early enough or at a threshold of test-and-isolate capacity of an assumed 16,000 incident cases per day, the course of the pandemic could have been controlled. An acute ramp-up of public health personnel at the beginning of the outbreak would have been a key sole success factor, reducing the need for other NPIs. The effect of resource-dependency is due to the fact that the β-reducing factor ω decreases reciprocally with the number of cases, making “test-and-isolate” a very strong intervention for small case numbers — and literally useless for very high case numbers.

Our results show that social distancing is a strong contributor to overall efficacy. Furthermore, social distancing measures may need to last for months to effectively control transmission and mitigate the possibility of resurgence (Lai et al. 2020). Social distancing comprises a wide range of changes, such as change in greeting rituals (no hand/body contact), sneezing etiquette, contact reduction to meeting only two households, and minimising contact when infected or having been close to someone being infected. Kisser et al. assumed a social distancing efficacy on reducing R ranged between 0 and 60% (Kissler et al. 2020). Data from the 1918 influenza pandemic in the United States confirms that being implemented at an early phase of the epidemic resulted in a lower death peak and a trend toward lower cumulative excess mortality (Hatchett et al. 2007). One-time social distancing efforts may push the SARS-CoV-2 pandemic peak into autumn and winter, whereas intermittent social distancing might maintain critical care demand within current thresholds. However, in order to achieve the latter a widespread surveillance of measures might be required to exactly time the distancing measures and avoid overshooting critical care capacity. (Kissler et al. 2020).

Analysing the course of the pandemic retrospectively with lifting-off of those NPIs being of lower agreed impact (i.e., “hand hygiene”, “close restaurants”, and “close schools”), the resulting peak in simultaneously infected cases would have reached about 1 million cases. This less restrictive mitigation approach would presumably not have overburdened the German healthcare system, as the number of German COVID-19 patients requiring intensive care unit beds (ICU) did not exceed 3000 beds per day at any time during the course of the pandemic, with a corresponding reserve of more than 5000 free ICU beds always having been available (Deutsche Interdisziplinäre Vereinigung für Intensiv- und Notfallmedizin (DIVI) 2020).

On a prospective basis, after gradually lifting off NPIs in summer only a full coming-into-force of all NPIs would have been able to fully contain spread of SARS-COV-2 again. When keeping “test-and-isolate” with reinforcing “keeping distance”, “wearing protection masks”, “hand hygiene”, and “ban of large public events”, the estimated number of infected new cases is in the range 2 million real cases (having applied an underreporting factor of 5). When assuming a share of 15% COVID-19 patients developing severely ill states and a subgroup of 10% requiring intensive care, the capacity of 30,000 ICU beds would have been sufficient (Kluge et al. 2020; Lee et al. 2020). However, acknowledging the high testing rate in Germany resulting in a higher share of less severely affected patients and when assuming population-based hospitalization rates from the US Center of Disease Control of 4.7 per 100,000 infected cases, only a third of the full capacity would have been needed (Garg et al. 2020). Furthermore, our prevalence estimates are based on an underreporting rate of 5, whereas reported critical care resource utilization rates are based on officially reported figures. Hence, capacity of the German critical care resources would even be more sufficient, allowing for further flexibility in choosing appropriate NPIs.

Our model has several limitations. It does not account for demographics, heterogeneities in contact and mobility patterns, spatial effects, stochasticity, inhomogeneous mixing, severity of disease, and reinfection with COVID-19.

Furthermore, seasonal effects are still unclear, as well as the reinfection rate, and were not part of the current modelling approach. Our model might be subject to overestimation as well as underestimation due to an insufficient understanding of the nature of outbreaks, i.e., incorrectly accounting for imported cases and outbreaks arising in subpopulations with higher transmission rates (Mercer et al. 2011).

A limitation could be the rather high estimate of compliance of test and isolate; however, in the first wave during 2020 this expert estimate was consistent with rather high estimates by public health departments and RKI. In addition, we assumed an initial test and isolate capacity of public health departments in Germany of 1000 incident cases per day to be managed, contacts to be analysed and to be followed up for at least 2 weeks. Based on figures recently published by the public health department of Bingen, with additional personnel and scouts being recruited, an average capacity of 5000 new identified cases per day can be derived in the third quarter of 2020 and should be applied in future modelling scenarios and in the public domain version (Schmidt et al. 2020).

**Fig. 8:**
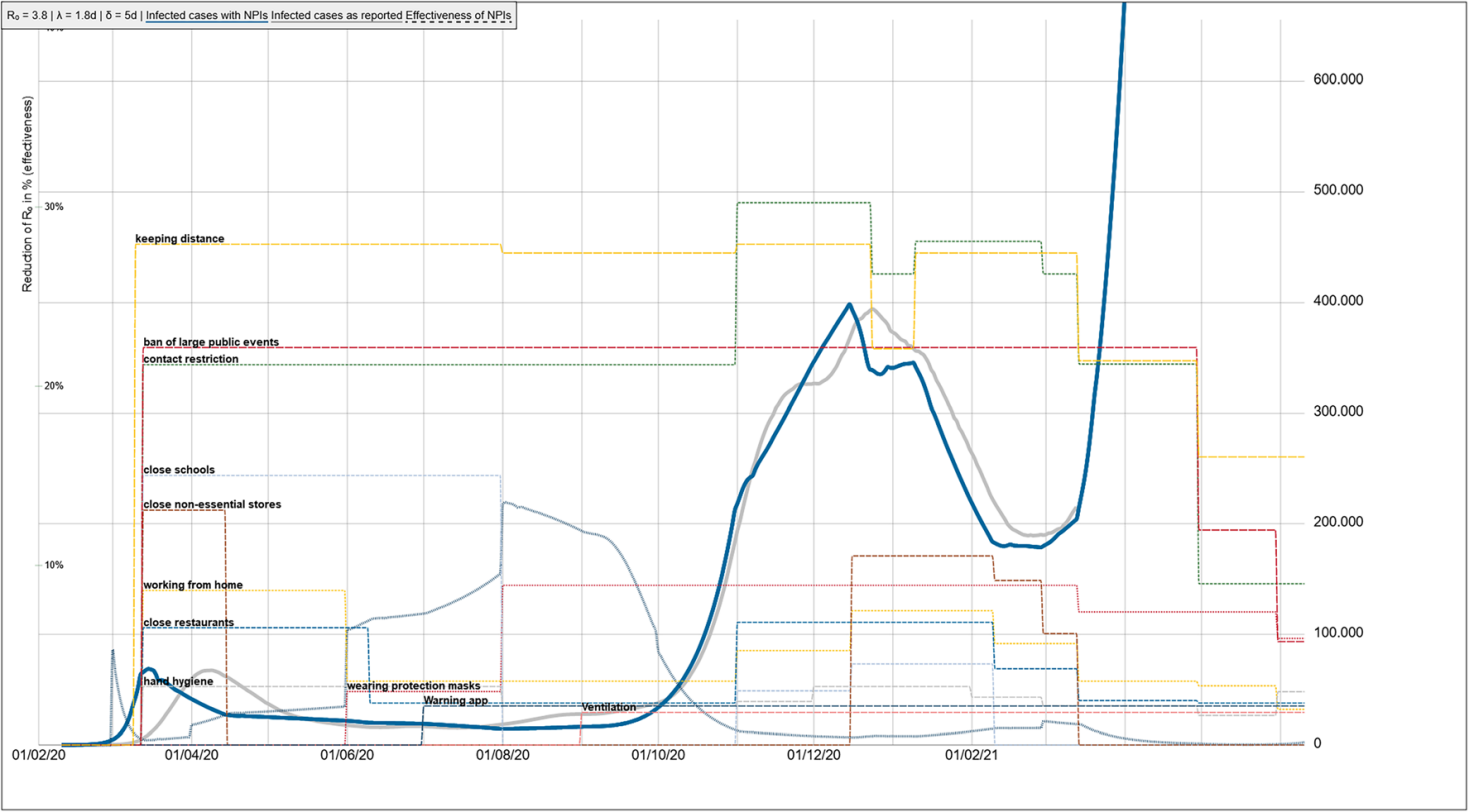
Sensitivity analysis for basic reproduction number R_0_ and latency period. *R*_0_: basic reproduction number, *λ*: latency period, *δ*: duration of infectiousness, NPI: non-pharmaceutical interventions. *Blue line*: simulated case numbers, *grey line*: case numbers as reported, *dotted lines*: effectiveness of NPIs**.** Left *y*-scale — effectiveness of measures; right *y*-scale *—* population

A further limitation of a sophisticated journal review process is the delay in publishing modeling results of real-time relevance. A more recent development of the model was able to highlight the need for a relevant shut-down beginning of 2021 in order to mitigate a second wave (Fig. 8). However, even with an increased test capacity of the public health department to an average capacity of 5000 new identified cases per day, lifting NPI measures in March would result in a third wave due to the still high amount of susceptibles without immunity.

Infection rate is the product of an average transmission probability and the average number of contacts per time observed. We initially planned for differentiating between the two latter factors; however, we realized that real-world contact rates for specific target populations are not readily available for Germany. We see a need to expand modelling of the efficacy contribution of NPIs on a more granular level, requiring further sociological studies with regard to, for example, contact behaviour in different population subgroups in the future.

Beyond the current model remit, we see a need to also differentiate the nature of measures: Bi et al. show that contact-based interventions are more efficient than case-based interventions to reduce transmission, since infected contacts are typically isolated earlier in their infection history than index cases (Bi et al. 2020). However, this approach would require an increase in capacity of public health departments in Germany.

## Conclusion

Employing an evidence-educated Delphi-panel approach for generating effectiveness estimates of non-pharmaceutical interventions (NPI) is feasible and could possibly help to generate model simulations with close replication of reported infected cases in Germany. Future curbing scenarios require a combination of NPIs. A Delphi-panel-based NPI assessment and modelling might support public health policy decision-making by informing sequence and number of needed public health measures. Our transparent interactive and publicly available model could help to inform policy decisions.

## Supplementary Information

ESM 1 (DOCX 147 kb)

## Data Availability

Access to model on request.

## Footnotes

1 Reported range of R_0_ 1.5–6.7 according to Liu et al.

2 About half of infected cases occurred in incubation period, hence assumption was selected below incubation period of 3 or 4 days reported by Ni and colleagues.

3 Infectiousness persisted for 10 days after symptom onset, however half of infected cases occurred in incubation period, hence 5 days were chosen.

4 AHA is the abbreviation for the German words “Abstand”, “Hygiene” and “Alltagsmasken” (i.e., “keeping distance”, “hand hygiene” and “wearing protection masks”)

## Code availability (software application or custom code)

Access to code on request.
