# Supplementary material for: Impact of public health interventions to curb SARS-CoV-2 spread assessed by an evidence-educated Delphi panel and tailored SEIR model
Source: Z Gesundh Wiss. 2021 May 17;31(4):539–52. doi: 10.1007/s10389-021-01566-2 (PMC8127459; doi:10.1007/s10389-021-01566-2)
Supplement: Supplementary file 1 — (DOCX 147 kb) [file 10389_2021_1566_MOESM1_ESM.docx]

# Supporting Information Details

To:

Impact of public health interventions to curb SARS-CoV-2 spread assessed by an evidence-educated Delphi-panel and tailored SEIR model

# Methods

## Model specific information

We employ a discrete, deterministic SEIR model on a daily basis for a period of less than one year without considering crude or Covid-19 specific death rates.(Kermack and McKendrick 1927) The transition from exposed to infectious and from infectious to recovered state is supposed not to follow an exponential random process (with rates 1/ λ resp. 1/δ ) as in standard SEIR models with N(t) = β(t) I(t) S(t) / P being the number of newly infected individuals (P = size of the population)


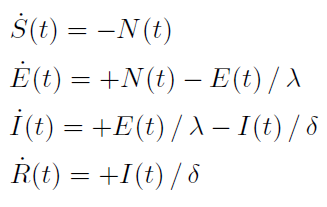


but to occur after constant times λ (the latency period) and λ + δ (the duration of infectedness, with δ being the duration of infectiosity) after infection. Our SEIR model thus is a system of delay differential equations:


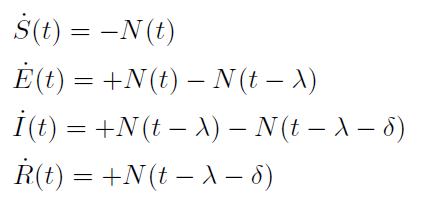


To be able to cope with non-integer latency periods and durations of infectiosity we used


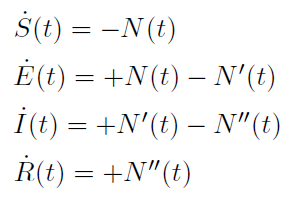


with


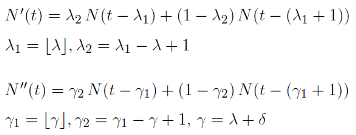


The effectiveness of non-pharmaceutical intervention measures was modelled as a temporary reduction of the (basic) transmission rate βₒ. We distinguished between *scalable measures* that do not depend on the number of infected individuals and *resource-dependent measures* that depend on a given capacity (e.g. number of test-and-isolate per day) divided by the number of infected individuals I(t) which are those accessible by the measure.

The combination of all individual NPI measures could theoretically reach 100% resulting in the extinction of the pandemic course. However even in a setting where a wide range of NPIs had been implemented comprehensively and restrictively like in China an extinction could not be observed.(Bi et al. 2020; Lai et al. 2020) Hence, we addressed a potential bias in overestimating combined effectiveness of NPIs as follows: Let for any NPI measure μ_i_ ω_i_ be its maximal effectiveness and ϕ_i_(t) be the time-dependent compliance to it with both ω_i,_ ϕ_i_(t) ∈ [0,1]. For a scalable measure the infection rate βₒ is reduced by a factor 1 - ω_i_ ϕ_i_(t). For a capacity dependent measure μ_i_ with capacity κ_i_(t) the infection rate βₒ is reduced by a factor 1 - ω_i_ ϕ_i_(t) κ_i_(t) / I(t). For a set of NPI measures the effectiveness of the measures multiply:


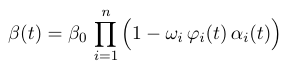


with αi(t) = 1 for scalable measures and αi(t) = κi(t) / I(t) for resource-dependent measures. We approximated a global scaling factor Φ for all compliances ϕi(t) in order to achieve a fit with reported data and changed it by the same relative amount:


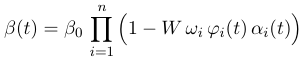


For each NPI we imputed an officially announced coming into force date (either obtained on federal level or if not available approximated via Länder information), an estimated advanced uptake period of the NPI, a decline of compliance after reaching maximum compliance as well as a spill-over effect of each NPI addressed as a residual-compliance > 0 after the announced coming out of force date.

The following base case assumptions were chosen for obtaining best fit with reported data:

- Rₒ = 3.8(Liu et al. 2020b) ^[[1]](#footnote-1)^
- latency period λ = 1.8 days (Guan et al. 2020; Jing et al. 2020; Liu et al. 2020a) ^[[2]](#footnote-2)^
- duration of infectiousness δ = 5 days (Jing et al. 2020; Singanayagam et al. 2020) ^[[3]](#footnote-3)^
- infection rate βₒ = Rₒ/ δ = 0.76/day
- share of immunity α= 15% of the total population
- estimated ratio of unreported cases = 5
- reporting delay = 8 days.

All scalable NPIs except “test-and-isolate” are employing a function of gradually being introduced, waning out over time except for “ban of large events”. All time points for NPI measure initiation, NPI measure duration and NPI measure relief can be changed individually. The NPI “test-and-isolate” was considered to be resource-dependent according to the resource being available at public health departments in the first quarter of 2020. We estimated the minimum initial capacity for the “test and isolate” strategy in February based on available medical personnel of local and regional public health departments resulting in a capacity of 1000 incident cases per day in Germany to be approached, checked for further contacts and followed up.

# Modelling relevant Discussion topics

The impact of pre-symptomatic and asymptomatic transmission is not fully considered in modelling according to some authors.(Sun and Viboud 2020) Due to COVID-19's nature of shedding via asymptomatic cases also, we consider incorporating a latency period into a COVID-19 model as reasonable. Based on the finding by Liu et al. our model differentiates between being infected but not infectious (during the latency period) and being infectious.(2017; Liu et al. 2020c) Different from recently published models, where the rate of transition from infected to infectious state is 1/ λ and where the rate 1/ δ determines the transition from infectious to recovered state(Dehning et al. 2020), we apply the latency period λ and the duration of infectiousness δ as constant times, after which the transitions take place, i.e. with λ days resp. λ + δ after infection.(Madore 2020)

We assumed a latency period of 1.8 days as asymptomatic infections at time of laboratory confirmation has been reported from many settings (ECDC 2020) with a subgroup of 16% remaining asymptomatic.(Byambasuren et al. 2020) Jing and colleagues found that patients with COVID-19 were at least as infectious in the incubation periods as during their illness periods.(Jing et al. 2020) Oher modelling results informed that with control measures in place pre-symptomatic transmission contributed to 48% and 62% of transmissions in Singapore and China, resp..(Ganyani et al. 2020) This is in line with findings that the viral load profile of SARS-CoV-2 is similar to that of influenza with a peak at around the time of symptom onset.(ECDC 2020)

Several models recently published, such as Davies et al. expand the number of measures being scrutinized. Subject of debate is the issue to calculate virus shedding also on the number of underlying contacts in different risk and age groups which other authors considered not feasible.(Davies et al. 2020) We did not stratify according to risk however focused on a wider range of NPIs, a more differentiated timing of NPIs and a differentiation in capacity restricted and non-capacity restricted measures. In addition to the impact of school closures, physical distancing, self-isolation of symptomatic individuals, and a combination of all four we simulated in addition personal protection masks, hand hygiene, test and isolate, ban of large public events, working from home and closure of restaurants as well as non-essential stores. We excluded shielding of older people (i.e., ≥70 years) as from a political point of view we considered this as ethically not acceptable.

Our model shows sensitivity to changes in Rₒ assumptions. This sensitivity is in line with other authors concluding that moderate changes in contact behaviour correspond to dramatically different evolutionary trajectories for Rₒ on longer time scales.(Lin et al. 2016) With the currently applied base case value of 3.8 we obtain predicted cases close to the officially reported German data. However, when discussing potential impact of measures with German public health experts a high pre-measure adherence was assumed due to the wide media coverage of drastic situations in other European countries.

The magnitude of a pandemic is often based on the size of the Rₒ.(Heffernan et al. 2005) Defining, calculating, interpreting, and applying Rₒ is not straightforward.(Delamater et al. 2019) Furthermore, applying Rₒ from other regions is restricted due to differing sociobehavioural and environmental factors.(Ridenhour et al. 2014) This might be particularly true for superspreading events such as après ski parties in Ski resorts and carnival occasions, which were initiating elements of the German pandemic.(Streeck et al. 2020) Whereas average reported initial Rₒ for Germany were in the range of two and three (RKI) we assumed that after successfully erasing the first Webasto-cluster a considerable initiation of the pandemic situation in Germany was due to travellers returning from vacation, particularly skiing vacation. Here, a considerable amount was infected(Gudbjartsson et al. 2020; Knabl et al. 2020), and a nationwide spread of multiple spots were initiated. Hence, as the key issue for the interpretation of Rₒ values is the period for which an estimate is valid, (Ridenhour et al. 2014) we assumed an initial Rₒ > 3 being more appropriate.

Development of Rₒ over time in our model corresponds with data from Shenzen, China, where the mean Rₒ substantially reduced from a baseline non-intervention value of 2.0–4.0 mean Rₒ in Shenzhen to a very low value at 0.41.(Sun and Viboud 2020) Bi et al. show that for a given R, the longer the infectious period, the more easily the epidemic can be brought under control with case-based interventions, which could be observed also in our modelling results.(Bi et al. 2020)

We adjusted the effectiveness contribution of each estimated measure proportionally meeting a reported overall measure effectiveness. Maximum overall effectiveness was assumed not to exceed a ceiling threshold of joint effectiveness of all measures of 80 %, which is in line with measures being reported in China: With intensive control measures, the community Shenzhen was able to reduce the effective reproduction number.(Bi et al. 2020)

### Strengths

Rₒ is supposed to be an indicator of the contagiousness or transmissibility of infectious and parasitic agents and is perceived to inform whether an outbreak is expected to continue (if Rₒ >1) or to end (Rₒ<1).(Delamater et al. 2019) However, Breban et al. have proven that population-level predictions based upon an ODE model that use the Rₒ value found by contact tracing as a threshold parameter may be inaccurate.(Breban et al. 2007) They strongly suggest that only an epidemic threshold parameter can be used to design control strategies via fitting an ODE model to population-level data. Our model is applying this approach whilst using Rₒ based SEIR modelling as well as the Delphi-panel informed simulation of measure effectiveness in order to fit the model to the German population-level data.

Our model assumptions are also conservative with regard to the assumed underreporting rate.(Li et al. 2020)

Furthermore, our model interface is characterized by an easy to use GUI, which allows for fast change of underlying assumptions by expert groups.

### Limitations

Couto et al. stated that residential mobility restriction presented itself as the most effective measure. The SEIR model associated with mobility parameters proved to be a useful tool in determining the chance of COVID-19 outbreak control.(Couto et al. 2020) However, to our knowledge these data are not readily available and would therefore in itself require a research activity, which normally is not available in an acute pandemic related decision setting. Hence, we did not incorporate, but found nevertheless reliable prognostic value.

Virus transmission is influenced by contact rate and a COVID-19 characteristic probability of transmission.(Lin et al. 2016) Both factors are affected by the presence of symptoms and disease severity, e.g. increased symptom severity will decrease contact rate but increase probability of transmission if a contact take place.(Lin et al. 2016) In order to model this specific issue further empirical data on how illness affects contact patterns are required. As these data are still not readily available also our model is based on a less granular simulation of disease transmission with a changing Rₒ due to selected NPI measures taken over time.

Furthermore, we are not modelling the impact of duration of infectiousness without symptoms, which might contribute to viral shedding as case reports confirmed asymptomatic transmissions with a recently reported share of up to four fifths of infected cases being asymptomatic.(Day 2020) However, a large proportion of asymptomatic cases developed some symptoms at a later stage of infection.(Cereda et al. 2020)

T, Li Y, Wang F, Zhou C (2017) Nonadherence to sublingual immunotherapy in allergic rhinitis: a real-life analysis Int Forum Allergy Rhinol 7:389-392 doi:10.1002/alr.21909

Bi Q et al. (2020) Epidemiology and transmission of COVID-19 in 391 cases and 1286 of their close contacts in Shenzhen, China: a retrospective cohort study Lancet Infect Dis doi:10.1016/S1473-3099(20)30287-5

Breban R, Vardavas R, Blower S (2007) Theory versus data: how to calculate R0? PLoS One 2:e282 doi:10.1371/journal.pone.0000282

Byambasuren O, Cardona M, Bell K, Clark J, McLaws M-L, Glasziou P (2020) Estimating the extent of asymptomatic COVID-19 and its potential for community transmission: systematic review and meta-analysis medRxiv:2020.2005.2010.20097543 doi:10.1101/2020.05.10.20097543

Cereda D et al. (2020) The early phase of the COVID-19 outbreak in Lombardy, Italy arXiv

Couto B et al. (2020) Mobility Restrictions For The Control Of Covid-19 Epidemic SciELO - Scientific Electronic Library Online

Davies NG, Kucharski AJ, Eggo RM, Gimma A, Edmunds WJ, Centre for the Mathematical Modelling of Infectious Diseases C-wg (2020) Effects of non-pharmaceutical interventions on COVID-19 cases, deaths, and demand for hospital services in the UK: a modelling study Lancet Public Health 5:e375-e385 doi:10.1016/S2468-2667(20)30133-X

Day M (2020) Covid-19: four fifths of cases are asymptomatic, China figures indicate BMJ 369:m1375 doi:10.1136/bmj.m1375

Dehning J, Zierenberg J, Spitzner FP, Wibral M, Neto JP, Wilczek M, Priesemann V (2020) Inferring change points in the spread of COVID-19 reveals the effectiveness of interventions Science 369 doi:10.1126/science.abb9789

Delamater PL, Street EJ, Leslie TF, Yang YT, Jacobsen KH (2019) Complexity of the Basic Reproduction Number (R0) Emerg Infect Dis 25:1-4 doi:10.3201/eid2501.171901

ECDC (2020) Transmission of COVID-19.

Ganyani T, Kremer C, Chen D, Torneri A, Faes C, Wallinga J, Hens N (2020) Estimating the generation interval for COVID-19 based on symptom onset data medRxiv:2020.2003.2005.20031815 doi:10.1101/2020.03.05.20031815

Guan WJ et al. (2020) Clinical Characteristics of Coronavirus Disease 2019 in China N Engl J Med 382:1708-1720 doi:10.1056/NEJMoa2002032

Gudbjartsson DF et al. (2020) Spread of SARS-CoV-2 in the Icelandic Population N Engl J Med 382:2302-2315 doi:10.1056/NEJMoa2006100

Heffernan JM, Smith RJ, Wahl LM (2005) Perspectives on the basic reproductive ratio J R Soc Interface 2:281-293 doi:10.1098/rsif.2005.0042

Jing QL et al. (2020) Household secondary attack rate of COVID-19 and associated determinants in Guangzhou, China: a retrospective cohort study Lancet Infect Dis doi:10.1016/S1473-3099(20)30471-0

Kermack WO, McKendrick AG (1927) A Contribution to the mathematical theory of epidemics Proceedings of the Royal Society of London 115:701-721 doi:10.1007/BF02464423

Knabl L et al. (2020) High SARS-CoV-2 Seroprevalence in Children and Adults in the Austrian Ski Resort Ischgl medRxiv:2020.2008.2020.20178533 doi:10.1101/2020.08.20.20178533

Lai S et al. (2020) Effect of non-pharmaceutical interventions to contain COVID-19 in China Nature doi:10.1038/s41586-020-2293-x

Li R, Pei S, Chen B, Song Y, Zhang T, Yang W, Shaman J (2020) Substantial undocumented infection facilitates the rapid dissemination of novel coronavirus (SARS-CoV-2) Science 368:489-493 doi:10.1126/science.abb3221

Lin CJ, Deger KA, Tien JH (2016) Modeling the trade-off between transmissibility and contact in infectious disease dynamics Math Biosci 277:15-24 doi:10.1016/j.mbs.2016.03.010

Liu D et al. (2020a) Real-Time Forecasting of the COVID-19 Outbreak in Chinese Provinces: Machine Learning Approach Using Novel Digital Data and Estimates From Mechanistic Models J Med Internet Res 22:e20285 doi:10.2196/20285

Liu Y, Gayle AA, Wilder-Smith A, Rocklov J (2020b) The reproductive number of COVID-19 is higher compared to SARS coronavirus J Travel Med 27 doi:10.1093/jtm/taaa021

Liu Z, Magal P, Seydi O, Webb G (2020c) A COVID-19 epidemic model with latency period Infect Dis Model doi:10.1016/j.idm.2020.03.003

Madore D, Alexander (2020) Exact solutions and analysis of an SIR variant with constant-time recovery.

Ridenhour B, Kowalik JM, Shay DK (2014) Unraveling R0: considerations for public health applications Am J Public Health 104:e32-41 doi:10.2105/AJPH.2013.301704

Singanayagam A et al. (2020) Duration of infectiousness and correlation with RT-PCR cycle threshold values in cases of COVID-19, England, January to May 2020 Euro Surveill 25 doi:10.2807/1560-7917.ES.2020.25.32.2001483

Streeck H, Hartmann G, Exner M, Schmid M (2020) Vorläufiges ergebnis und schlussfolgerungen der covid-19 case-cluster-study (Gemeinde Gangelt). <https://www.b1.ro/pictures/documents/1237-zwischenergebnis_covid19_case_study_gangelt_0.pdf>. Accessed 1.8.2020 2020

Sun K, Viboud C (2020) Impact of contact tracing on SARS-CoV-2 transmission Lancet Infect Dis doi:10.1016/S1473-3099(20)30357-1

1. Reported range of R0 1.5 – 6.7 according to Liu et al. [↑](#footnote-ref-1)
2. About half of infected cases occurred in incubation period, hence assumption was selected below incubation period of 3 or 4 reported by Ni and colleagues. [↑](#footnote-ref-2)
3. Infectiousness persisted for 10 days after symptom onset, however half of infected cases occurred in incubation period, hence 5 days were chosen. [↑](#footnote-ref-3)
